# Ginsenosides ameliorates high altitude-induced hypoxia injury in lung and kidney tissues by regulating PHD2/HIF-1α/EPO signaling pathway

**DOI:** 10.3389/fphar.2024.1396231

**Published:** 2024-07-18

**Authors:** Peng Ji, Zepeng Zhang, E. Mingyao, Qing Liu, Hongyu Qi, Tong Hou, Daqing Zhao, Xiangyan Li

**Affiliations:** ^1^ Key Laboratory of Active Substances and Biological Mechanisms of Ginseng Efficacy, Jilin Provincial Key Laboratory of Bio-Macromolecules of Chinese Medicine, Northeast Asia Research Institute of Traditional Chinese Medicine, Ministry of Education, Changchun University of Chinese Medicine, Changchun, Jilin, China; ^2^ Research Center of Traditional Chinese Medicine, College of Traditional Chinese Medicine, Changchun University of Chinese Medicine, Changchun, Jilin, China

**Keywords:** total ginsenoside, high-altitude hypoxia, oxidative stress, inflammation, PHD2/HIF-1α/EPO

## Abstract

**Background:** The primary constituent of ginseng, known as ginsenosides (GS), has been scientifically demonstrated to possess anti-fatigue, anti-hypoxia, anti-inflammatory, and antioxidant properties. However, the effect and mechanisms of GS on tissue injury induced by high-altitude hypoxia still remain unclear.

**Aim of the study:** This study aims to investigate the protective effect of GS on a high-altitude hypoxia model and explore its mechanism.

**Materials and methods:** Sprague-Dawley rats were placed in a high-altitude simulation chamber for 48 h (equivalent to an altitude of 6,000 m) to establish a high-altitude hypoxia model. We assessed the anti-hypoxic efficacy of GS through blood gas analysis, complete blood count, and hemorheology analysis. We used H&E and hypoxia probe assays to evaluate the protective effect of GS on organ ischemia-induced injury. Further, we used ELISA and qPCR analysis to detect the levels of inflammatory factors and oxidative stress markers. Immunohistochemistry and immunofluorescence staining were performed to determinate protein expression of hypoxia inducible factor 1-alpha (HIF-1α), erythropoietin (EPO), and prolyl hydroxylase 2 (PHD2).

**Results:** In the survival experiment of anoxic mice, 100 mg/kg of GS had the best anti-anoxic effect. GS slowed down the weight loss rate of rats in hypoxic environment. In the fluorescence detection of hypoxia, GS reduced the fluorescence signal value of lung and kidney tissue and alleviated the hypoxia state of tissue. Meanwhile GS improved blood biochemical and hematological parameters. We also observed that GS treatment significantly decreased oxidative stress damage in lung and kidney tissues. Further, the levels of inflammatory factors, IL-1β, IL-6, and TNF-α were reduced by GS. Finally, GS regulated the PHD2/HIF-1α/EPO signaling pathway to improve blood viscosity and tissue hyperemia damage.

**Conclusion:** GS could alleviate high-altitude induced lung and kidney damage by reducing the level of inflammation and oxidative stress, improving blood circulation through the PHD2/HIF-1α/EPO pathway.

## Graphical Abstract

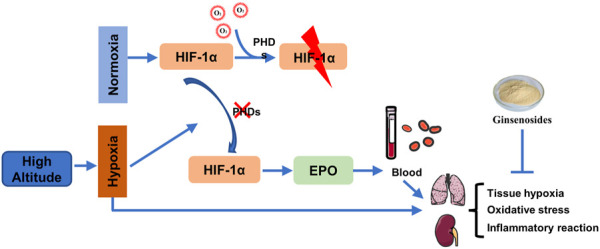

## 1 Introduction

High altitude hypoxia results from decrease in atmospheric pressure and in consequence in atmospheric PO_2_. Hypobaric hypoxia triggers a wide range of responses. It is mainly characterized by symptoms such as difficulty breathing, chest tightness, cyanosis, and reduced physical endurance (Swenson and Bartsch, 2012). This condition typically occurs within 2 days of exposure to altitudes above 2,500 m, causing reactions that, if not promptly addressed, can result in irreversible harm to tissues and circulation (Bartsch and Swenson, 2013). The pathological process of hypoxia at high altitudes in a rapidly progressing plateau environment involves a reduction in ventilation due to increased altitude, leading to compensatory erythrocytosis within the body. As hypoxic stress intensifies, noticeable hypoxemia and hypocapnia occur, ultimately resulting in tissue and organ damage (Sikter et al., 2017). Currently, the main methods for dealing with high-altitude hypoxia include timely descent and oxygen therapy (Davis and Hackett, 2017).

Oxygen is the substrate that maintains normal function of various organs and cells in the body, and hypoxia at high altitudes can lead to accumulation of reactive oxygen species (ROS) (Jing et al., 2022). Excessive ROS levels lead to an increase in the pro-oxidant substance MDA (malondialdehyde), as well as a decrease in the antioxidant SOD (superoxide dismutase), GSH (glutathione reduced), and CAT (catalase), disrupting the oxidative balance within tissues and cells, ultimately resulting in oxidative stress (Pena et al., 2022). Hypoxia also promotes the production of various such as interleukin-1β (IL-1β), interleukin-6 (IL-6), tumor necrosis factor-alpha (TNF-α), nuclear factor kappa B (NF-κB), leading to tissue inflammation response (Pu et al., 2022).

*Panax* ginseng C. A. Meyer (P. ginseng), a traditional herb with extensive usage, enjoys immense popularity across the globe, particularly in Asian countries (Piao et al., 2020). The ginsenosides (GS) stand out as the most crucial and extensively researched active component found in ginseng. It exhibits diverse effects encompassing anti-inflammatory, antioxidant, anti-hypoxia, anti-fatigue properties alongside neuroprotective attributes (Ratan et al., 2021). *Rhodiola rosea, L*., also known as ‘Ginseng of the Plateau’ contains a major component called salidroside (Sal). This substance has effects such as anti-fatigue, anti-hypoxia, anti-aging, and immune regulation (Zhang et al., 2021).

In the hypoxic environment of high altitudes, there are changes in the levels of electrolytes and blood cell count, which affect the overall energy and nutrient transport in the body (Liu et al., 2023). The lungs are highly sensitive to changes in external environmental pressure and oxygen levels, while the kidneys, as one of the organs with abundant blood supply, also possess exceptional sensitivity in detecting oxygen (Xie et al., 2020). Under hypoxia, the activity of PHD2 is inhibited, inhibiting HIF-1a hydroxylation and allowing its accumulation in tissues, which in turn triggers downstream EPO protein expression. We propose that the presence of low oxygen levels in high-altitude areas may alter blood circulation through the PHD2/HIF-1α/EPO pathway, leading to the development of high-altitude diseases. The potential molecular mechanisms of GS in the treatment of tissue damage caused by high-altitude conditions require further investigation. This study aims to establish a rat model simulating hypoxia at high altitudes and evaluate the potential of GS as a treatment for this condition.

## 2 Materials and methods

### 2.1 Medicines and reagents

The ginsenosides (the purity >90%) from ginseng was prepared as previously reported (Zhang et al., 2023), which was prepared from dry roots of *Panax* ginseng (5-year-old, Changbai Mountain, Jilin, China) and contains ginsenoside Rb1, Rb2, Rb3, Rc, Rd, Re, Rg1, and other monomers. Salidroside was obtained from MedChemExpress (MCE, State of New Jersey, United States) The Hypoxyprobe Omni Kit was purchased from Hypoxyprobe, Inc (Massachusetts, United States). NF-κB-p65, HIF-1α, PHD2, and EPO antibodies were obtained from Santa Cruz Biotechnology (CA, United States). All other chemicals and reagents used in this study have commercially available standard biochemical quality.

### 2.2 Animals

Male BALB/c mice (SPF grade, weighing 18–22 g, 6–8 weeks) and male Sprague-Dawley (SD) rats (weighing 180–220 g, 6–7 weeks) were obtained from SPF Biotechnology (Beijing, China). All animals were accommodated at a temperature of 23°C ± 2°C, humidity of 50% ± 5%, and under a 12-h light-dark cycle, with free access to food and water. The experiments commenced after 1 week of acclimation. This experiment was approved by the Bioethics Committee of Changchun University of Chinese Medicine and the Institutional Animal Care (Approval No. 20190100), which was conducted based on the guideline for the use of laboratory animals.

### 2.3 Acute hypoxia mice model and drug treatment

Fifty BALB/c mice (male, SPF grade, 18–22 g) were separated into five groups (10 per group): Hypoxic control group (HC, physiological saline, 0.1 mL/kg), GS low-dose group (GS-L, 40 mg/kg), GS medium-dose group (GS-M, 100 mg/kg), GS high-dose group (GS-H, 160 mg/kg), and salidroside group (Sal, 100 mg/kg). After oral administration for 7 days with the last dose given 1 hour before the experiment, all mice were placed in a hypoxic chamber with an O_2_ concentration of 8% and observed for survival rate within 15 min (Shi et al., 2021).

### 2.4 Rat high-altitude hypoxia model and drug administration

Four groups were formed by randomly assigning twenty-four male SD rats: normoxia control group (NC, physiological saline, 1 mL/100 g), hypobaric hypoxia model group (HH, physiological saline, 1 mL/100 g), ginsenosides group (GS, 100 mg/kg), and Salidroside group (Sal, 100 mg/kg). Each group consisted of six rats. All rats were orally administered the respective substances for seven consecutive days. One hour after the last administration, the HH group, GS group, and Sal group were placed in an animal high-altitude simulation chamber. Based on the preliminary experiments, we gradually increased the altitude to 6,000 m above sea level at a speed of 10 m/s and maintained it for a duration of 48 h. The normoxia control group was kept under laboratory conditions with the same atmospheric pressure as sea level. During this period, food and water were provided normally. After the experiment, rats were administered 2% sodium pentobarbital (0.3 mL/100 g body weight) for anesthesia. Tissue and blood samples were then collected and preserved for subsequent analysis.

### 2.5 Lung water content (LWC) analysis

After 48 h of exposure to high-altitude hypoxia, the wet weight of the left lung in rats was measured using a precision electronic balance. Subsequently, the lung tissue was placed in an oven at 55°C for 72 h to obtain a constant dry weight. Lung water content (%) = (wet weight - dry weight)/wet weight × 100% (Pan et al., 2020).

### 2.6 Blood gas analysis

The rats were promptly anesthetized upon removal from the high-altitude simulation chamber. Blood samples extracted from the abdominal aorta and placed into anticoagulant tubes containing lithium heparin. The blood samples then transferred onto a biochip using a syringe. Alterations in acid-base equilibrium and electrolyte levels, encompassing pH, partial pressure of oxygen (PO_2_), partial pressure of carbon dioxide (PCO_2_), concentration of bicarbonate ions (HCO_3_^−^), base excess (BE), anion gap (AnGap), and blood oxygen saturation (SO_2_%) (Nehra et al., 2016).

### 2.7 Blood routine test

Following anesthesia of rats, blood samples were collected from the abdominal aorta using EDTA-K_2_ vacuum collection tubes. Subsequently, 300 μL whole blood samples were aspirated utilizing a pipette for subsequent analysis employing the ProCyte DX fully automated hematology analyzer. The components include red blood cells (RBC), hemoglobin (HGB), hematocrit (HCT), mean corpuscular volume (MCV), platelets (PLT), white blood cells (WBC), and neutrophils (NEUT).

### 2.8 H&E staining

After 48 h in a high-altitude simulation chamber, the right upper lobe of the lung and left kidney were dissected and fixed in 4% paraformaldehyde for 72 h. Following dehydration treatment using a graded ethanol series, the tissues were embedded in paraffin and sectioned into 3 μm-thick paraffin sections. The tissue sections were stained with hematoxylin and eosin (H&E) for histopathological examination under a light microscope (Wu et al., 2013). In the pathological examination of lung tissue, our primary focus lies in assessing the thickness of alveolar walls and evaluating the extent of inflammatory infiltration. Similarly, when examining kidney tissue, we primarily concentrate on observing morphological alterations in glomeruli as well as assessing the presence of inflammatory infiltration.

### 2.9 Oxidative stress analysis

Lung tissues and PBS were mixed in a ratio of 1:9 to prepare a 10% tissue homogenate. The mixture was centrifuged at 1,500 rcf, 4°C for 10 min, and the supernatant was collected for analysis. The activities of MDA (A003-1-2), SOD (A001-2-2), GSH (A006-2-1), and CAT (A007-1-1) in rat lung tissues were assessed using micro-reagent kits (NanJing JianCheng Bioengineering Institute) to analyze the oxidative stress response. The total protein content of the samples was determined using the BCA method.

### 2.10 Hypoxyprobe detection in multiple tissues

Animals were intraperitoneally injected with Hypoxyprobe at a dose of 60 mg/kg body weight. Tissues were collected at 30 min post-injection and fixed in 4% paraformaldehyde for 72 h, followed by paraffin embedding. The tissues were sectioned and deparaffinized, followed by rehydration, and then incubated overnight at 4°C with the hypoxia probe (rabbit anti-PAb2627AP, diluted 1:200). The hypoxia signal was visualized using a Hyper Fluor 488-conjugated goat anti-rabbit IgG antibody (diluted 1:500 from ApexBio) (Sun et al., 2016).

### 2.11 Quantitative PCR analysis

Tissue samples (15–20 mg) were added into lysis buffer (TianGen, Beijing, China) and total RNA extraction was performed using the TianGen kit with centrifugal column method. The total RNA was reverse transcribed into cDNA and analyzed using SYBR quantitative PCR (qPCR) with the following cycling conditions: 10 min of denaturation at 95°C, followed by 40 cycles of 30 s at 95°C and 30 s at 60°C. The data were normalized to β-actin and calculated using the 2^−ΔΔCt^ method. Primers were provided by Sangon Biotech Co., Ltd. (Shanghai, China). The contents of primer sequences are shown in Table 1.

**TABLE 1:** The primers for the quantitative real-time RT-PCR analysis.

Gene	Forword sequence (5′-3′)	Reverse sequence (3′-5′)
Rat IL-1β	AAT​CTC​ACA​GCA​GCA​TCT​CGA​CAA​G	TCC​ACG​GGC​AAG​ACA​TAG​GTA​GC
Rat IL-6	ACT​TCC​AGC​CAG​TTG​CCT​TCT​TG	TGG​TCT​GTT​GTG​GGT​GGT​ATC​CTC
Rat TNF-α	CAC​CAC​GCT​CTT​CTG​TCT​ACT​GAA​C	TGG​GCT​ACG​GGC​TTG​TCA​CTC
Rat β-actin	ACT​GCC​GCA​TCC​TCT​TCC​TC	AAC​CGC​TCG​TTG​CCA​ATA​GTG

### 2.12 Enzyme-linked immunosorbent assay

The levels of IL-1β, IL-6, and TNF-α in the tissue samples and EPO level in serum were quantified using the commercial ELISA kits (Boyan Biotech, Shandong, China), following the manufacturer’s instructions.

### 2.13 Immunohistochemistry staining

The lung and kidney tissue specimens were embedded in paraffin and consecutively sliced. The slices were baked at 65°C for 40 min, followed by dewaxing and hydration. The sections were then placed in EDTA buffer solution and heated for antigen retrieval. Endogenous peroxidase was blocked with 3% H_2_O_2_ for 10 min. At room temperature, the tissue on the slice was subjected to blocking with a 10% goat serum blocking solution for a duration of 30 min. Add primary antibodies to tissue slices and incubate overnight at 4°C. Subsequently, a second antibody conjugated with HRP targeting mice/rabbits was introduced and incubated at room temperature for 1 h. Finally, we applied DAB chromogenic solution for color development while carefully controlling the duration of DAB staining under a light microscope. After each step, the tissue sections were washed three times with PBS for 5 min. Subsequently, hematoxylin and eosin staining was performed on the sections, followed by sealing in neutral resin (Livingston et al., 2023). The antibodies used were as follows: HIF-1α (sc-13515, 1:300, Santa Cruz Biotechnology), PHD2 (sc-271835, 1:300, Santa Cruz Biotechnology), and EPO (sc-5290, 1:500, Santa Cruz Biotechnology). We selected three sections from each group, and images from six fields were captured from each section and averaged for semi-quantitative analysis.

### 2.14 Immunofluorescence staining

After embedding in OCT freezing medium and section cutting, tissue sections underwent antigen retrieval using EDTA buffer (pH = 8.0), and followed by washing with PBS three times. Subsequently, the sections were incubated in a blocking solution for 30 min and then kept overnight at 4°C with a primary antibody, NF-κB-p65 (1:300, sc-8008, Santa Cruz Biotechnology) diluted in PBS. Next, the slices were incubated with the fluorescent-dye conjugated second antibody, Alexa Fluor 594 (1:400, Yeasen 33212ES60) for 50 min, and stained with DAPI for 5 min, and then mounted with anti-fade fluorescence mounting medium (Wei et al., 2023). The fluorescent images were acquired by a fluorescence microscope (Leica GER) at ×20 magnification and the positive area was calculated semi-quantitatively by imajeJ.

### 2.15 Statistical analysis

All experimental data are presented as the mean ± SD. The differences among groups were analyzed using GraphPad Prism 9.0 (GraphPad Inc.) software, employing one-way ANOVA and Dunnet’s test. *p*-values *p* < 0.05 was as statistically.

## 3 Results

### 3.1 GS increased the survival rate of acute hypoxia-induced mice

We first conducted an acute hypoxia experiment to demonstrate the anti-hypoxia effect of GS as previous reported. As shown in Table 2, GS-L (30 mg/kg), GS-M (100 mg/kg), GS-H (160 mg/kg), and positive drug, Sal (100 mg/kg) effectively enhanced the survival rate of mice within 15 min acute hypoxia. The survival rates of these groups were 10%, 40%, 22%, and 20%, respectively. Therefore, we chose GS at the dose of 100 mg/kg as the intervention condition for subsequent experiments.

**TABLE 2:** Survival number and rate of acute hypoxia-induced mice in hypoxia, GS or Sal groups.

Groups	Number	Dose (mg/kg)	Survival number	Survival rate (%)
HC	10	0	0	0
GS-L	10	30	1	10
GS-M	10	100	4	40
GS-H	9	160	2	22
Sal	10	100	2	20

HC, normal oxygen control; GS, ginsenosides; Sal, salidroside.

### 3.2 GS enhanced the adaptation of rats to high altitude-induced acute hypoxia

**FIGURE 1:**
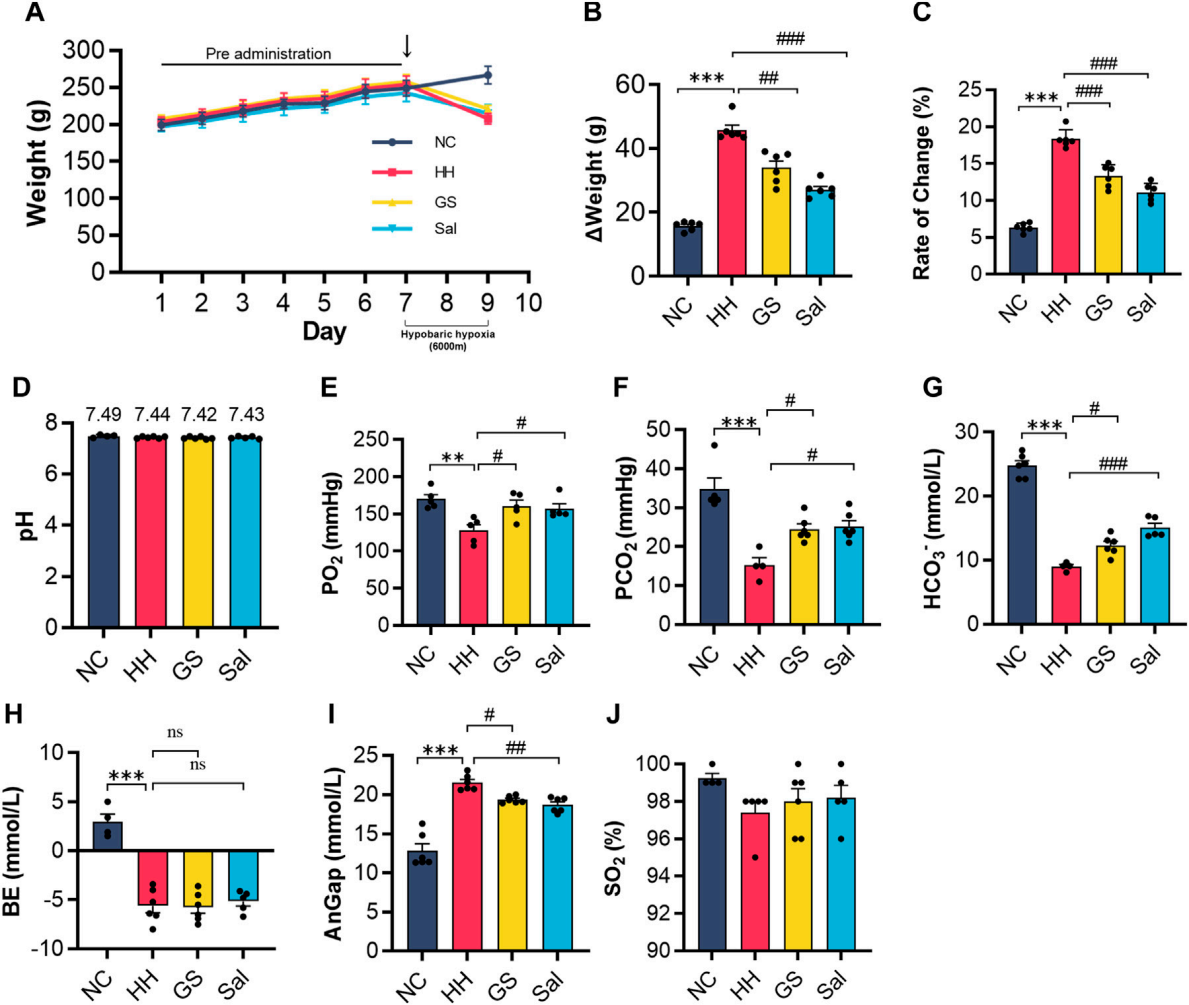
GS enhances the adaptability of rats to high-altitude environments. **(A)** The weight changes of rats from 7 days of pre administration to 48 h of modeling **(B,C)** The weight change and weight change rate of rats from day 7 to the end of modeling on day 9. The level of **(D)** pH, **(E)** PO_2_, **(F)** PCO_2_, **(G)** HCO_3_^−^, **(H)** BE, **(I)** AnGap, **(J)** SO_2_% in blood gas analysis. All data are presented as means ± SD (n = 6). **p < 0.01, ***p < 0.001 vs. NC group; #*p* < 0.05, ##*p* < 0.01, ###*p* < 0.001 vs. HH group. PO_2_, oxygen partial pressure; PCO_2_, partial pressure of carbon dioxide; HCO_3_^−^, bicarbonateion; BE, base excess; AnGap, anion gap; SO2%, blood oxygen saturation. NC: Normoxia Control, HH: Hypobaric Hypoxia, GS: Ginsenosides, Sal: Salidroside.

In order to ascertain whether GS can enhance can adaptability to high-altitude environments, we placed rats in a simulated environment at 6,000 m altitude for 2 days (from day 7 to 9). Body weight can generally be used as an indicator of high-altitude environmental adaptation. We found that high altitude exposure induced a dramatic weight loss of nearly 20% in rats. By contrast, GS and Sal pre-treatment shown a weight (Figures 1A–C). Another risk factor of high-altitude environment metabolic acidosis. The results of blood gases analysis showed after 48 h of hypoxia, PO_2_, PCO_2_, HCO_3_^−^ and BE were lower, and AnGap was higher, in the HH group compared to the NC group, whilst SO_2_% and pH did not change. (Figures 1E–J). Compared with the HH group, there was no significant change in pH (Figure 1D), while AnGap exhibited a significantly lower level in the GS and Sal groups (Figure 1I). Conversely, the values of PO_2_, PCO_2_, HCO_3_- showed a notable increase. The BE excess did not change (Figure 1H), when HH, GS and Sal are compared. In the GS and Sal intervention groups, there was no significant change in the levels of SO_2_% compared to the HH group (Figure 1J). These findings indicate that GS can induce changes in blood electrolyte levels (such as PO2, PCO2, HCO3 - and AnGap) to facilitate adaptation to high altitude.

### 3.3 GS improved arterial blood routine and blood viscosity in high altitude-induced rats

**FIGURE 2:**
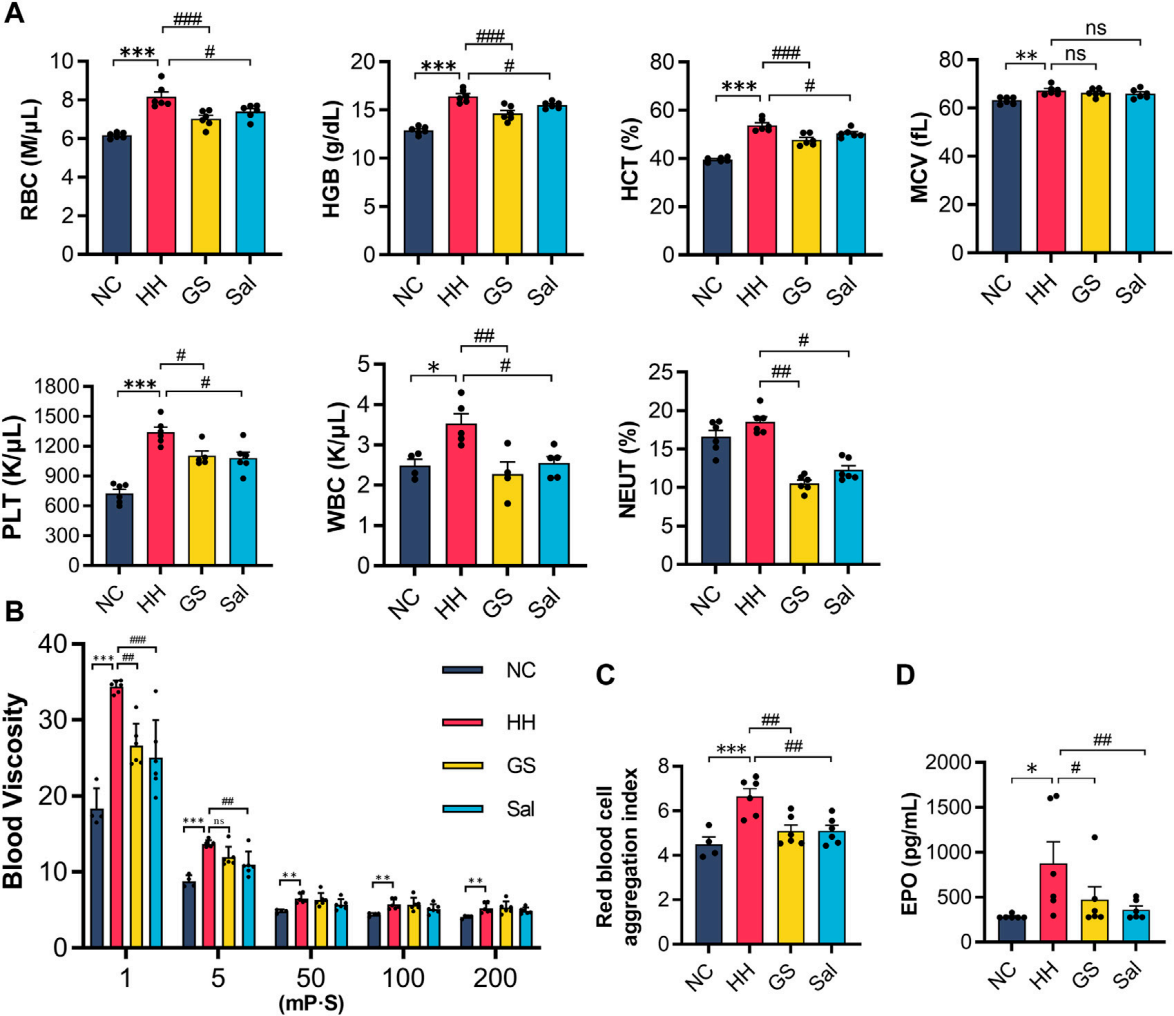
The effect of GS on arterial blood routine and blood viscosity in high-altitude hypoxic rats. **(A)** Blood routine examination. **(B)** Whole blood viscosity. **(C)** Red blood cell aggregation index. **(D)** Serum EPO protein levels were analyzed by ELISA. All data are presented as means ± SD (n = 6). *p < 0.05, **p < 0.01, ***p < 0.001 vs. NC group; ^#^ *p* < 0.05, ^##^ *p* < 0.01, ^###^ *p* < 0.001 vs. HH group. RBC, red blood corpuscles; HGB, hemoglobin; HCT, hematocrit; MCV, mean cell volume; PLT, platelets; WBC, white blood corpuscles; NEUT, neutrophil count; EPO, erythropoietin. NC: Normoxia Control, HH: Hypobaric Hypoxia, GS: Ginsenosides, Sal: Salidroside.

In high-altitude environments, hypoxia could affect the flowability of red blood cells and blood viscosity. The blood showed that the levels of RBC, HGB, HCT, MCV, PLT, WBC in the blood of rats exposed to high-altitude hypoxic environments were slightly higher than those of the NC group. However, there was no significant change in the level of neutrophils NEUT% (Figure 2A). Compared with the HH group, GS and Sal intervention reduced the number of RBC, HGB, HCT and WBC. In the blood viscosity test, the whole blood viscosity was increased in HH rats compared to NC rats. At a shear rate of 1, blood viscosity decreased in the GS and Sal groups, and at a shear rate of 5, blood viscosity decreased in the GS group. However, at high shear rates (50,100 and 200), there was no significant change in GS and Sal groups (Figure 2B). Compared with the NC group, the RBC aggregation index increased in the HH group, while it decreased in the GS and Sal groups compared with the HH group (Figure 2C). ELISA detection showed a significant increase in serum EPO levels in the HH group compared to the NC group (Figure 2D). The intervention of GS and Sal reduced blood viscosity, red blood cell aggregation index, and EPO levels. Therefore, the intervention of GS could improve blood cell flowability, reduced blood viscosity, and effectively inhibited further development of high-altitude hypoxia damage.

### 3.4 The effect of GS on hypoxia in various tissues of rats in high-altitude environment

**FIGURE 3:**
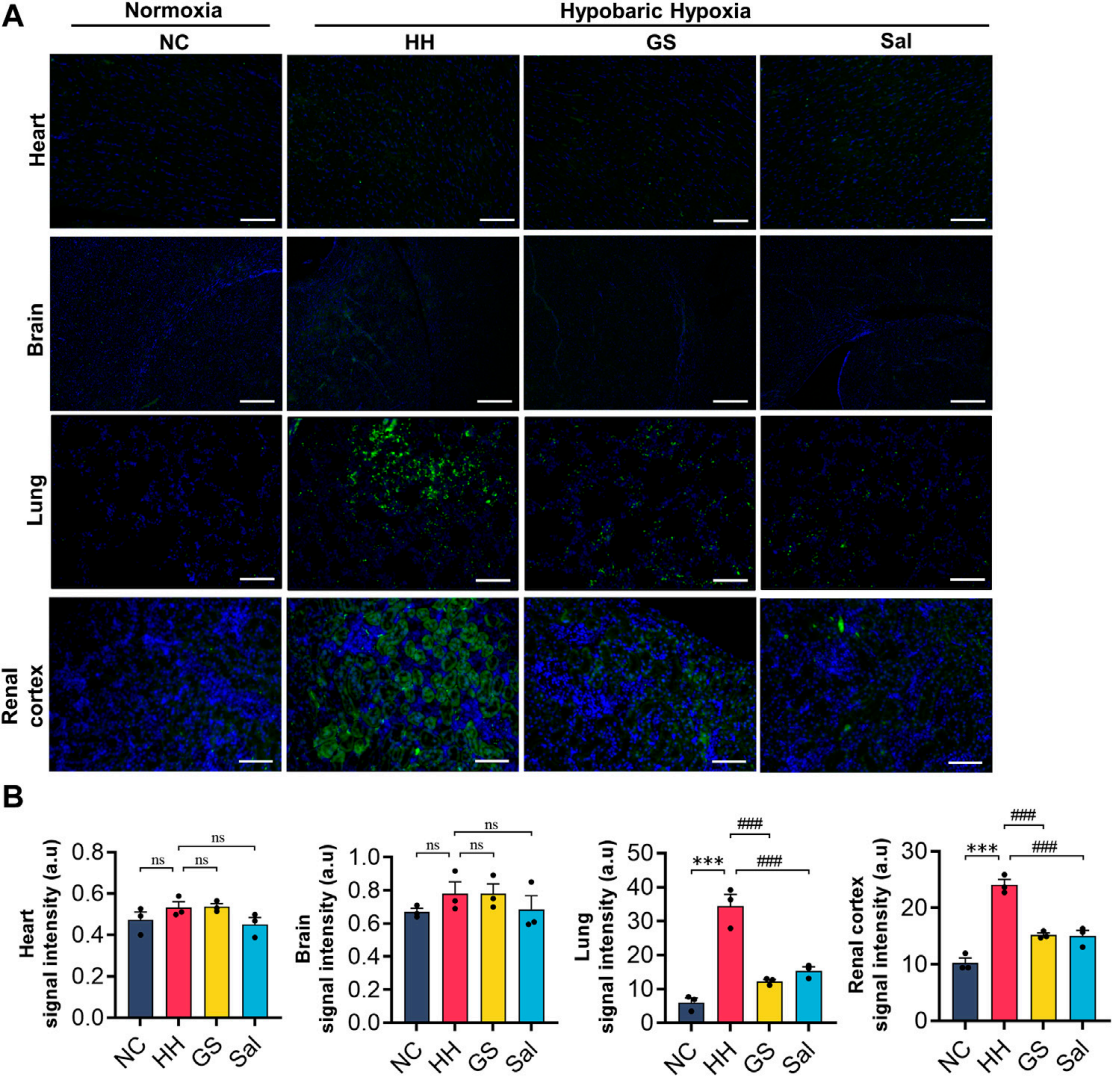
The effect of GS on hypoxia in various tissues of rats in high-altitude environment. **(A)** Hypoxia probes were used to measure hypoxia signals in rat heart, brain, lung, and kidney tissues (20×), scale bar = 50 μ m. **(B)** Quantification of hypoxia signals in various tissues. All data are presented as means ± SD (n = 6). ***p < 0.001 vs. NC group; ^###^ *p* < 0.001 vs. HH group. NC: Normoxia Control, HH: Hypobaric Hypoxia, GS: Ginsenosides, Sal: Salidroside.

To investigate the degree of hypoxia in various tissues of rats in a high-altitude environment, we used hypoxia probes to detect tissue hypoxia signals. The experimental results showed that no hypoxia probe signals were observed in the heart, brain, lung, and kidney tissue slices of the NC group rats. After 48 h of hypoxia treatment, we observed a significant increase in fluorescence intensity in the lung and kidney tissues of the HH group rats compared to the NC group, while there was no significant hypoxic signal in the heart and brain tissues (Figure 3A). On the contrary, the hypoxic signals in the lungs and kidneys of rats from the GS and Sal groups were significantly reduced compared to those in the HH group (Figure 3B), indicating that GS and Sal can effectively alleviate high-altitude-induced hypoxia in lung and kidney tissues.

### 3.5 GS ameliorated high altitude-induced lung injury

**FIGURE 4:**
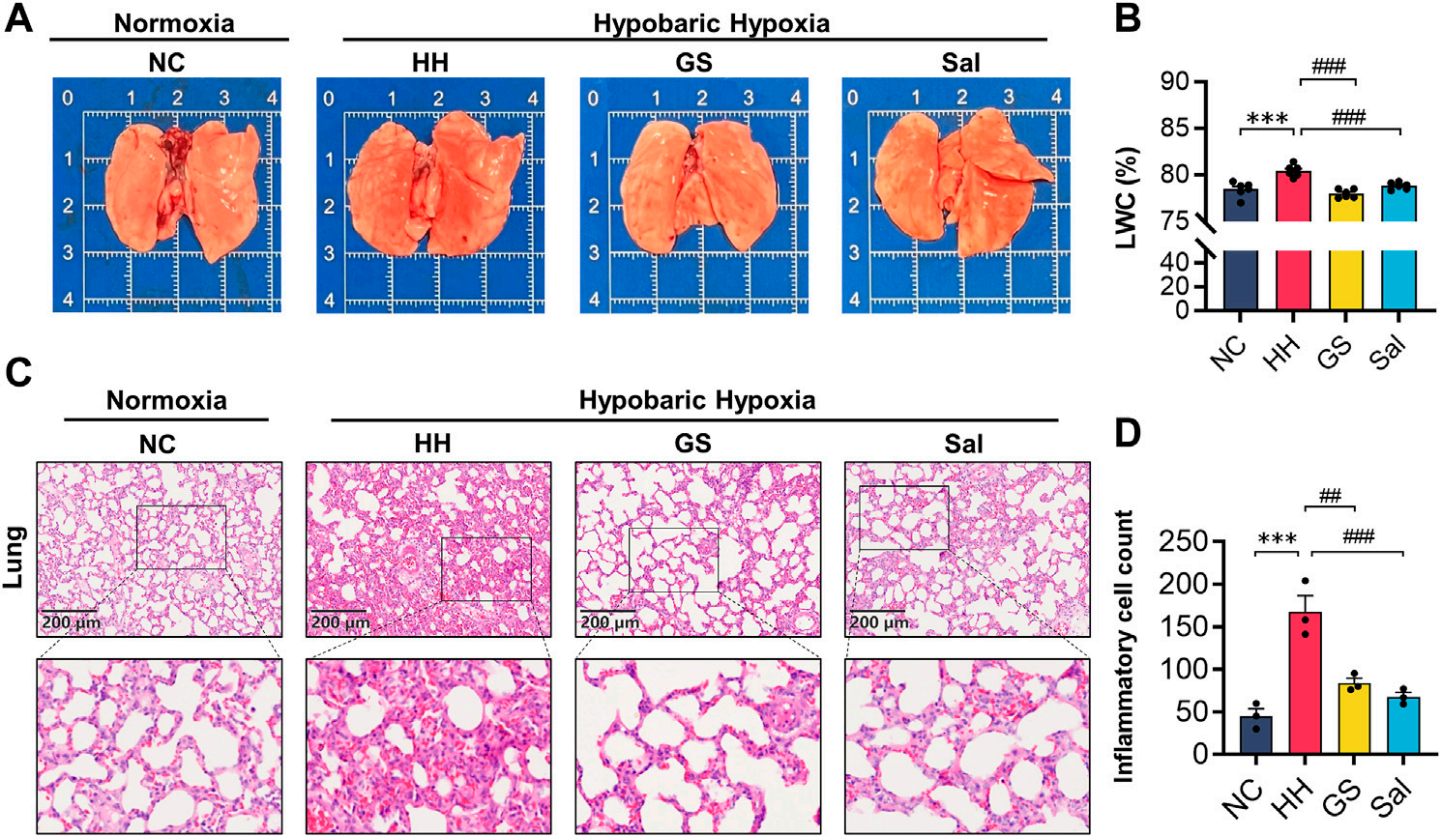
GS reduces lung injury in high-altitude hypoxic rats. **(A)** Morphology of lung organs in high-altitude hypoxic rats. **(B)** Lung water content (n = 6). **(C)** The H&E staining of rat lung tissue (10×), scale bar = 200 μ m. **(D)** Counting of inflammatory cells in lung tissue using H&E staining. All data are presented as means ± SD (n = 3). *p < 0.05, ***p < 0.001 vs. NC group; ^#^ *p* < 0.05, ^##^ *p* < 0.01, ^###^ *p* < 0.001 vs. HH group. NC: Normoxia Control, HH: Hypobaric Hypoxia, GS: Ginsenosides, Sal: Salidroside.

High altitude environments often induce pulmonary edema and hypoxia. We found the lungs of the HH group exhibited darker red color, increased volume, and noticeable dark spot damage in their morphology. These injuries by pre-treatment with GS and Sal (Figure 4A). Meanwhile, the LWC index in the HH group exhibited a significant exacerbation compared to the NC group following exposure to high-altitude. Rats pre-treated with GS (100 mg/kg) and Sal (100 mg/kg) exhibited a significant decrease of LWC (Figure 4B). Further, H&E staining and quantitative analysis of inflammatory cells in rat lung tissue showed that compared to the NC group, rats in the HH group exhibited pulmonary edema, thickening of alveolar septa accompanied by congestion, and inflammatory infiltration (Figures 4C, D). After GS and Sal pre-treatment, we observed a reduction in the degree of inflammation infiltration, significant improvement in congestion status and pathological morphology in the lung tissues of hypoxia-induced rats.

### 3.6 GS alleviated oxidative stress damage in high-altitude hypoxic rats

**FIGURE 5:**
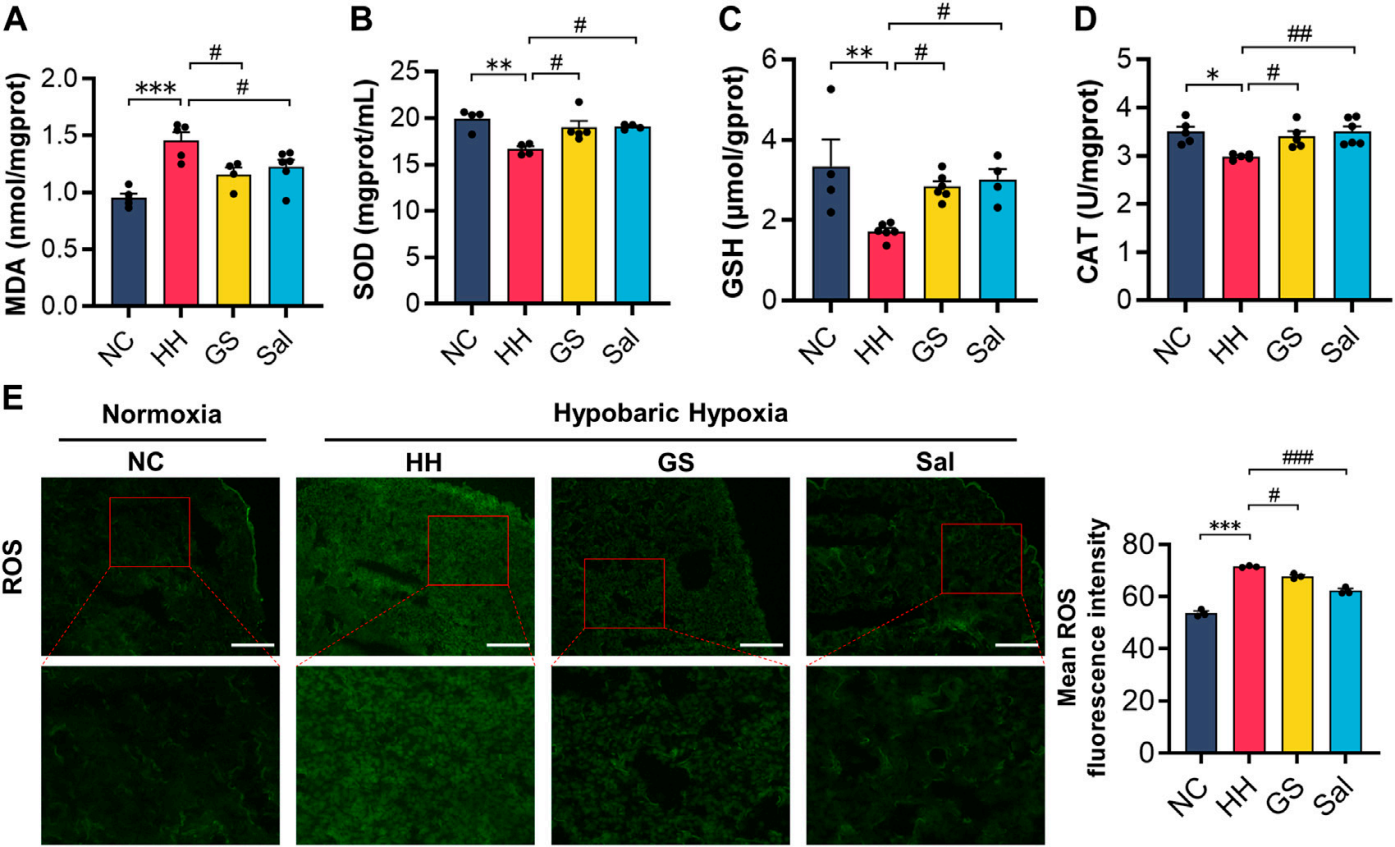
GS alleviates oxidative stress damage in high-altitude hypoxic rats. **(A–D)** The contents of MDA, SOD, GSH and CAT in lung tissue of rats **(E)** Immunofluorescence detection of ROS content in lung tissue of rats (n = 3). All data are presented as means ± SD (n = 6). *p < 0.05, **p < 0.01, ***p < 0.001 vs. NC group; ^#^ *p* < 0.05, ^##^ *p* < 0.01, ^###^ *p* < 0.001 vs. HH group. NC: Normoxia Control, HH: Hypobaric Hypoxia, GS: Ginsenosides, Sal: Salidroside.

To demonstrate whether GS can alleviate oxidative stress damage caused by high-altitude hypoxia, we measured the levels of oxidative markers in lung tissue. Our results found that the level of MDA in the HH group significantly increased, while the activities of SOD and CAT as well as the concentration of GSH decreased significantly compared to the NC group (Figures 5A–D). Similarly, compared to the NC group, the HH group exhibited an enhanced ROS fluorescence intensity in rat lung tissues (Figure 5E). However, compared with the HH group, GS and Sal showed significant inhibitory effects on the formation of oxidants MDA and ROS. At the same time, GS and Sal play a promoting role in the synthesis of SOD, GSH, and CAT, achieving an equilibrium in oxidation levels. These results confirmed that GS can alleviate the oxidative stress imbalance in lung tissue caused by high-altitude hypoxia.

### 3.7 GS reduced lung tissue inflammation damage in high-altitude hypoxic rats

**FIGURE 6:**
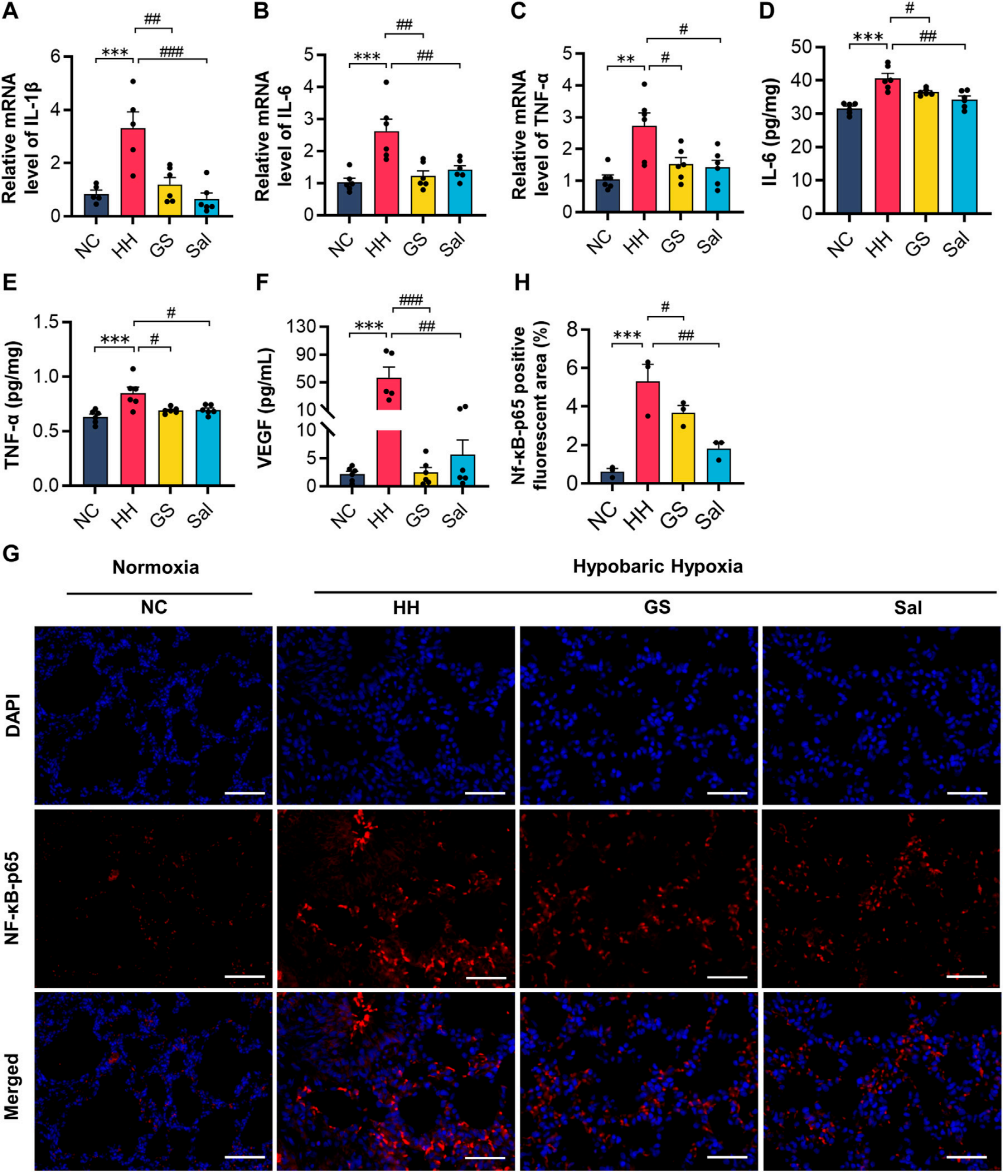
GS reduces inflammatory damage in high-altitude hypoxic rats. **(A–C)** The mRNA expression levels of IL-1β, IL-6 and TNF-α were analyzed by qPCR method. **(D–F)** The protein expression levels of IL-6, TNF-α and VEGF were analyzed by ELISA kits. All data are presented as means ± SD (n = 6). **(G)** Immunofluorescence staining of NF-κB-p65 (20×), scale bar = 50 μ m. **(H)** Positive fluorescent cell area of NF-κB-p65. (n = 3) *p < 0.05, **p < 0.01, ***p < 0.001 vs. NC group; ^#^ *p* < 0.05, ^##^ *p* < 0.01, ^###^ *p* < 0.001 vs. HH group. NC: Normoxia Control, HH: Hypobaric Hypoxia, GS: Ginsenosides, Sal: Salidroside.

Tissue damage caused by high-altitude hypoxia is often accompanied by reactive inflammation. Therefore, we further conducted tests on some inflammatory factors in lung tissues. The mRNA expression levels of IL-1β, IL-6, and TNF-α in lung tissue were analyzed using quantitative PCR technology. After exposure to hypoxia under low pressure, the mRNA expression levels of IL-1β, IL-6, and TNF-α were significantly increased in the HH group compared to the NC group (Figures 6A–C). The protein levels of IL-6 and TNF-α detected by ELISA were consistent with the above changes (Figures 6D–F). Meanwhile, the serum VEGF protein levels in the HH group were significantly higher than those in the NC group (Figure 6F). However, compared with the HH group, the GS and Sal pre-treatment groups significantly reduced the expression of these inflammatory factors. Furthermore, we examined the expression level of NF-κB-p65 by immunofluorescence staining (Figure 6G). In the HH group, the positive fluorescence area of NF-κB-p65 was significantly higher than that in the NC group, while it decreased in the GS and Sal groups compared to the HH group (Figures 6G, H). The findings suggested that GS and Sal can suppress hypoxia-induced inflammatory factors and alleviate inflammation reactions caused by high-altitude hypoxia.

### 3.8 GS improved hypoxic kidney injury in rats

**FIGURE 7:**
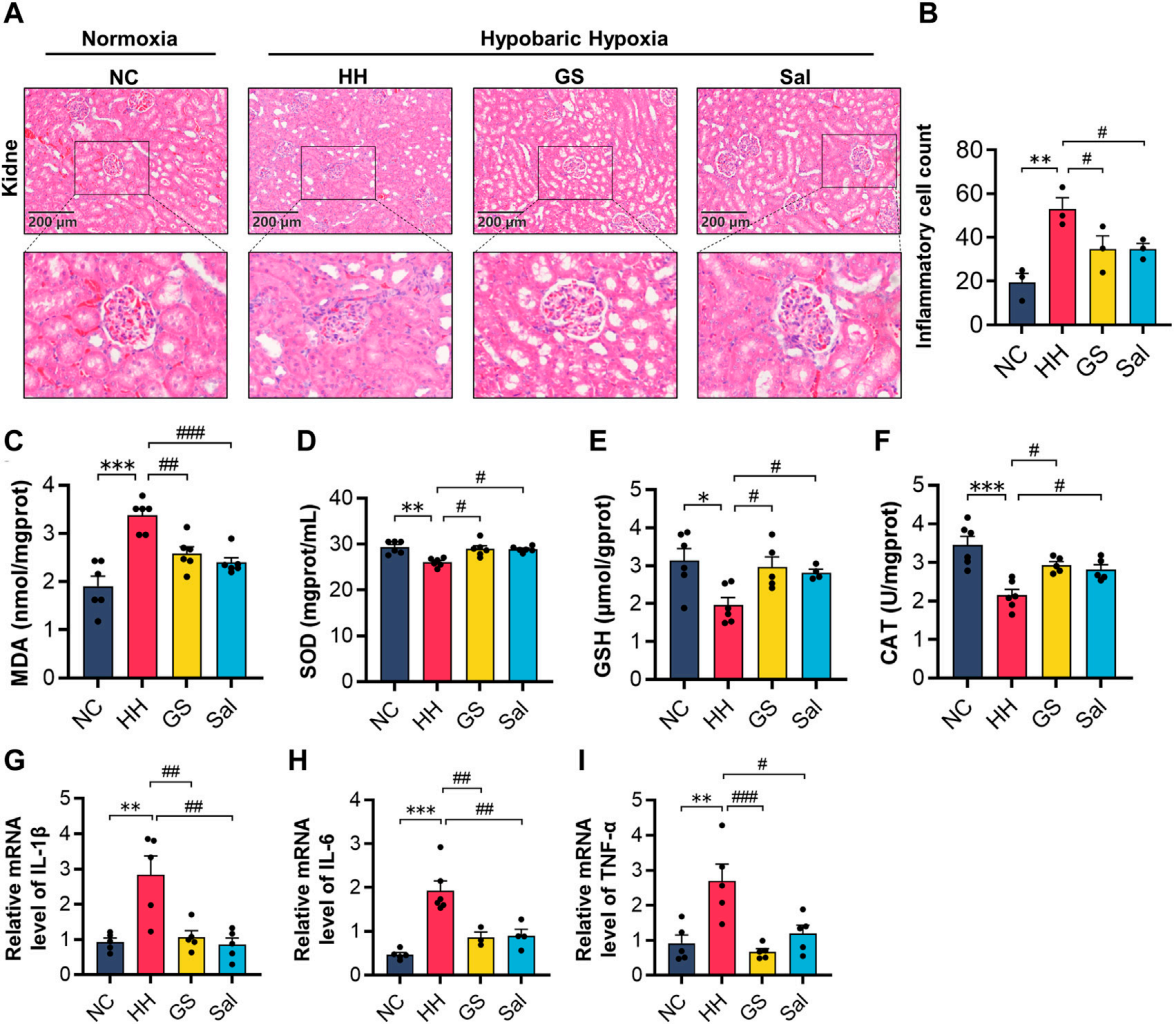
GS improves hypoxic kidney injury in rats. **(A)** The H&E staining of rat kidney (10×), scale bar = 200 μm. **(B)** Counting of inflammatory cells in renal tissue using H&E staining. **(C–F)** The contents of MDA, SOD, GSH and CAT in kidney tissue of rats. **(G–I)** The mRNA expression levels of IL-1β, IL-6, and TNF-α were analyzed by qPCR in kidney tissue of rats. All data are presented as means ± SD (n = 6). *p < 0.05, **p < 0.01, ***p < 0.001 vs. NC group; ^#^ *p* < 0.05, ^##^ *p* < 0.01, ^###^ *p* < 0.001 vs. HH group. NC: Normoxia Control, HH: Hypobaric Hypoxia, GS: Ginsenosides, Sal: Salidroside.

The kidneys are highly sensitive to hypoxia. Next, we conducted a study on high-altitude hypoxia-induced renal injury. In the H&E staining of the kidney, compared with the NC group, the HH group mainly showed mesangial cell proliferation, thickening of basement membrane, and renal tubular edema (Figure 7A). Quantitative analysis of inflammatory cells showed that the number of inflammatory cells in the HH group was higher than that in the NC group. Compared with the HH group, both GS and Sal pre-treatment groups had a significant reduction in the number of inflammatory cells (Figure 7B). Meanwhile, we investigated the impact of high-altitude hypoxia on oxidative stress in rat kidney tissue. The production of MDA in the HH group increased, while the other hand, both GS and Sal treatment effectively restored the changes in oxidative products and antioxidant enzymes in rat kidney tissues caused by high-altitude hypoxia (Figures 7C–F). Then, we further investigated the anti-inflammatory properties of GS in renal tissue using PCR. The results showed that HH significantly increased the mRNA levels of IL-1β, IL-6, and TNF-α, while GS and Sal decreased the expression of these pro-inflammatory factors, indicating that both of them possesses strong anti-inflammatory effects (Figures 7G–I). GS and Sal can improve renal tissue damage in rats under hypoxic condition.

### 3.9 GS ameliorated hypoxic injury in lung and kidney tissues via PHD2/HIF-1α/EPO pathway

**FIGURE 8:**
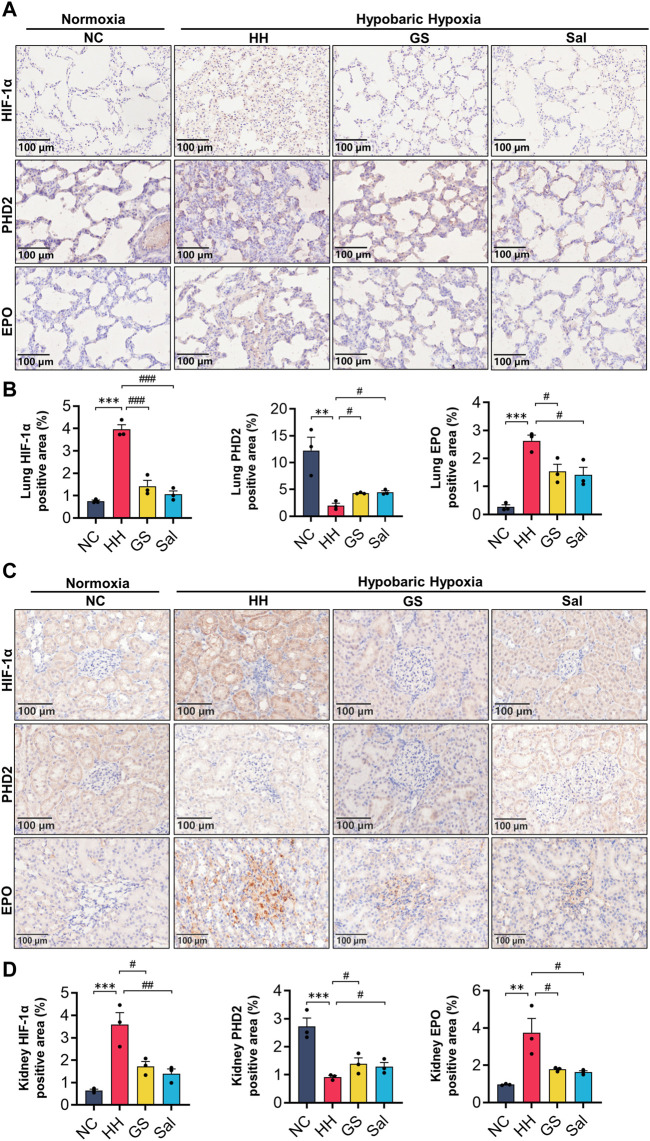
GS affects the expression of PHD2/HIF-1α/EPO pathway in lung and renal. **(A)** Immunohistochemical staining of PHD2, HIF-1α, and EPO proteins in lung tissue (20×), scale bar = 100 μm. **(B)** Quantification of EPO, HIF-1a, and PHD2 positive cell areas in lung tissue. **(C)** Immunohistochemical staining of PHD2, HIF-1α, and EPO proteins in renal tissue (20×), scale bar = 100 μ m. **(D)** Quantification of EPO, HIF-1a, and PHD2 positive cell areas in renal tissue. The three lung or kidney tissue samples from each group were used and the average values from six image fields in each sample were analyzed and showed. All data are presented as means ± SD (n = 3). **p < 0.01, ***p < 0.001 vs. NC group; ^#^ *p* < 0.05, ^##^ *p* < 0.01, ^###^ *p* < 0.001 vs. HH group. NC: Normoxia Control, HH: Hypobaric Hypoxia, GS: Ginsenosides, Sal: Salidroside.

To further investigate the relationship between hypobaric hypoxia and blood changes, we examined the expression of PHD2, HIF-1α, and EPO proteins in lung and kidney tissues. The results showed that compared with the NC group, HH induction increased the expression of HIF-1α nuclear protein and EPO protein in the lung and kidney tissues of rats, while decreasing the expression of PHD2 protein. After GS and Sal intervention, the expression of HIF-1α nuclear protein and EPO protein in the lung and kidney tissues was decreased, while the expression of PHD2 protein was increased. (Figures 8A–D). However, the differential expression of PHD2 and EPO proteins induced by HH in the lungs was smaller compared to renal tissue (Figures 8A, B). These results indicate that GS primarily regulates the expression of renal PHD2/HIF-1α/EPO proteins, affecting tissue hypoxic injury.

## 4 Discussion

High-altitude hypoxia typically occurs in environments above 2,500 m and was influenced by various factors (Luks et al., 2017). In this study, we investigated the potential protective effects of GS on high-altitude hypoxia-induced lung and kidney tissue damage as well as its underlying mechanisms in blood circulation. Our findings demonstrated that GS mitigated weight loss in rats exposed to high-altitude hypoxic conditions and regulated their blood electrolyte balance. Moreover, GS effectively attenuated lung and kidney injury caused by high-altitude hypoxia by suppressing oxidative stress and inflammatory responses in these tissues. Additionally, GS modulated the hemorheological characteristics of rats under high-altitude hypoxic environment, leading to reduced blood viscosity, enhanced oxygen delivery efficiency, and improved development of hypoxia symptoms.

LWC was an important indicator of lung injury and edema caused by high altitude hypoxia (Su et al., 2004; Sun et al., 2008). We observed a significant increase in lung water content in rats treated with HH. Additionally, the lungs showed thickened and congested alveolar interstitium, along with extensive inflammatory exudation. Electrolytes were an essential buffering system in maintaining the body’s acid-base balance (Pei et al., 2023). In high-altitude hypoxic environments, the electrolyte balance of blood is affected, specifically manifested by changes in pH value and a decrease in levels of bicarbonate ions and carbon dioxide in the body (Zouboules et al., 2018; Caldwell et al., 2021). The lack of oxygen in the body simultaneously caused anaerobic metabolism, acidosis, and imbalances in acid-base equilibrium, which also impacted tissue functionality (Galley and Webster, 1999).

Previous studies have shown that in acute low-pressure hypoxic environments, lung pathological features are inevitably associated with inflammation, oxidative stress response, and other factors (Bartsch et al., 2001; Faiss et al., 2013). During acute low-pressure hypoxia, the body’s ROS levels and oxidative stress damage were activated (Korde et al., 2011). The enzymes SOD, GSH, and CAT in the human body act as antioxidant defenders (Aguilar et al., 2018). The exposure of rats to a low-pressure hypoxic environment at 7,620 m altitude for different durations significantly increased lung levels of ROS and MDA, while decreasing SOD and GSH levels (Sarada et al., 2008). Our research found that GS and Sal pre-treatment corrected the oxidative imbalance caused by high-altitude hypoxia and reduced the damage caused by oxidative stress in lung and kidney tissues.

A previous study showed that NF-κB is widely recognized as an inflammatory inducer that drives the occurrence of inflammatory responses in the body during high-altitude hypoxia (Taylor, 2008). This process promoted the production of IL-1β, IL-6 and TNF-α, triggering an inflammatory response (Li and Verma, 2002). However, we observed only a change in the total fluorescence value for NF-κB-p65, not a significant change in the level of nuclear transcription. We only observed that both GS and Sal significantly reduced the levels of IL-1β, IL-6, and TNF-α. The expression levels of these factors significantly increased after exposure to HH. These results were consistent with the above research. Therefore, GS and Sal have reduced the level of inflammation caused by HH and played a crucial role in alleviating tissue inflammation damage.

Any inflammation was accompanied by changes in blood composition, especially under low-pressure hypoxia, which caused changes in blood viscosity and affected tissue oxygen supply (Raberin et al., 2022). Excessive red blood cell production may result in decreased blood flow velocity, increased viscosity, and reduced oxygen delivery efficiency (Franke et al., 2013). The study of Tibetan people revealed a faster blood flow rate, ensuring adequate oxygen supply to tissues and organs effectively (Peng et al., 2011). In addition, there was a close relationship between inflammation and blood, and the Blood routine testing played an important reference role in the diagnosis of high-altitude diseases. Previous studies showed that RBC, HGB, and WBC in rats exposed to high-altitude hypoxia for 24 h were significantly elevated (Sarada et al., 2015; Luan et al., 2019). The significant increase in red blood cells and hemoglobin after entering high altitude a compensatory response of the body (Li et al., 2018). However, this compensatory response may lead to permanent harm to the respiratory function and tissue (Mangin, 2017). As with previous studies, our research observed an increase in RBC, HGB, and HCT levels in rats exposed to high-altitude hypoxia. In addition, we also noticed a positive correlation between elevated blood EPO levels and elevated blood viscosity. After pre-treatment with GS and Sal, it was observed that the compensatory increase in blood cell count and the increase in blood viscosity in high-altitude hypoxic rats were effectively reduced.

Excessive production of red blood cells reduced blood flow velocity, increased viscosity, and consequently affected tissue oxygen supply efficiency. The expression of EPO played a crucial role in regulating the number of red blood cells and blood flow velocity. A study suggested that EPO protein was produced by the kidneys during HH induction, thereby affecting the generation of red blood cells in the blood (Frede et al., 2011). The renal blood flow was high and was exceptionally sensitive to changes in oxygen partial pressure, making it susceptible to hypoxia damage (Eckardt et al., 2003). Under HH induction, renal tubules and glomeruli underwent pathological morphological changes. A study confirmed that hypoxia caused an increase in HIF-1α accumulation in renal tubules and glomeruli. The regulation of EPO expression was intriguingly mediated by HIF-1α, while PHD2 played a pivotal role in governing the protein levels of both EPO and HIF-1α (Percy et al., 2007). HIF-1α played a crucial role as a factor regulated by hypoxia (Hu et al., 2003). Hypoxic conditions enhanced the expression of HIF-1α, which in turn induced the expression of specific genes such as EPO and VEGF (Eckardt et al., 2003). PHD2 (Egln2) an oxygen-sensitive rate-limiting enzyme (Berra et al., 2003). In a normoxic environment, PHD2 maintain low levels of HIF-1 α. However, under hypoxic conditions, the restricted activity of PHD2 due to limited oxygen availability led to nuclear translocation and accumulation of HIF-1α in tissues (Percy et al., 2007; Sharma et al., 2022).

In our study, we confirmed through immunohistochemistry that the accumulation of HIF-1α in the lung and kidney tissues of high-altitude hypoxic rats is negatively correlated with the expression of PHD2. In addition, as the expression of renal EPO increases, the levels of EPO in the serum of high-altitude hypoxic rats also significantly rise. Further hemorheological tests demonstrated that in high-altitude environments, the body’s blood was in a state of high viscosity, which affected oxygen delivery efficiency and further exacerbated tissue hypoxia. Under the intervention of GS and Sal, the expression of HIF-1α and EPO proteins in the kidneys of high-altitude hypoxic rats decreased, while the activity of PHD2 protein relatively increased. In the blood, intervention with GS and Sal decreased blood viscosity, ensuring tissue oxygen supply efficiency. With the suppression of inflammation and oxidative stress damage, it further alleviates tissue hypoxia symptoms in high-altitude hypoxic rats, to some extent preventing their further deterioration. Firstly, our experiment only demonstrates the changes in tissue damage and blood circulation in a high-altitude hypoxic environment, further evidence is needed to establish the specific relationship between the two. Furthermore, although we have explored the changes in lung tissue injury and blood under high-altitude hypoxic conditions, as well as various characteristics of GS in alleviating high-altitude hypoxia damage, the specific therapeutic targets and mechanisms of GS still need to be further investigated in future studies.

## 5 Conclusion

In summary, GS had a protective effect on lung and kidney tissue damage induced by high-altitude hypoxia. The mechanism of GS may be related with the improvement of blood circulation, and the reductions of tissue hypoxia, oxidative stress damage, and inflammatory response through the PHD2/HIF-1α/EPO pathway. Therefore, GS could have great potential in alleviating high-altitude discomfort and treating high-altitude diseases in the future.

## Data Availability

The original contributions presented in the study are included in the article/Supplementary Material, further inquiries can be directed to the corresponding authors.
